# Clinical effectiveness of a multidisciplinary quality improvement initiative to prevent nasal pressure injuries associated with nasotracheal tube: a historical controlled study

**DOI:** 10.3389/fmed.2026.1744744

**Published:** 2026-04-08

**Authors:** Ying Zhou, Yu Xue Wu, Kai Li, Chao Zhang, Lin Fan

**Affiliations:** 1 The Affiliated Stomatological Hospital of Chongqing Medical University, Chongqing, China; 2 Chongqing Municipal Health Commission Key Laboratory of Oral Biomedical Engineering, Chongqing, China; 3 Chongqing Key Laboratory of Oral Diseases, Chongqing, China; 4 Chongqing Municipal Key Laboratory of Oral Biomedical Engineering of Higher Education, Chongqing, China

**Keywords:** medical device-related pressure injury, multidisciplinary, nasal pressure injury, nasotracheal intubation, prevention

## Abstract

**Purpose:**

This study aimed to evaluate the impact of a multidisciplinary quality improvement initiative on reducing nasal pressure injuries related to nasotracheal tube (NTT) in patients undergoing prolonged oral, head and neck surgery.

**Methods:**

Transnasal intubation is frequently employed in these procedures to optimize surgical field exposure. With the growing complexity and duration of surgeries, particularly for malignant tumors, the risk of nasal pressure injuries has increased. These injuries contribute to greater pain, extended hospital stays, higher treatment costs, and delayed recovery, underscoring the need for effective preventive measures. Under the Hospital Patient Safety Initiative, a multidisciplinary team was established to implement a comprehensive prevention strategy. This involved structured risk assessment, dynamic and graded nursing interventions, and systematic postoperative follow-up. A historical control study was conducted, comparing nasal pressure injury incidence over two 12-month periods: pre- and post-intervention.

**Results:**

A total of 515 patients were included—221 in the historical control (HC) group and 294 in the intervention group (IG). Baseline characteristics were well-balanced between the two groups, with all standardized mean differences (SMD) below 0.1. The incidence of nasal pressure injuries (PI) significantly decreased from 7.24% in the HC group to 2.38% in the IG group (*p* = 0.008, two-sided). The absolute risk reduction (ARR) was 4.86% (95% CI: 1.19–9.23%), corresponding to a relative risk (RR) of 0.33 (95% CI: 0.14–0.79) and a number needed to treat (NNT) of 21 (95% CI: 11–85). Notably, stage 2 injuries were eliminated in the intervention group (3 vs. 0 cases), and no injuries of stage 3 or higher were observed in either cohort.

**Conclusion:**

The multidisciplinary bundle, featuring a modified CORN scale and graded interventions, effectively reduces the risk of device-related nasal pressure injuries. This approach is scalable for perioperative nursing management in prolonged head and neck surgeries.

## Introduction

Medical Device Related Pressure Injury (MDRPI) is a pressure injury (PI) caused by sustained contact of a medical device with the skin or subcutaneous tissues, resulting in impaired blood circulation, which in turn causes tissue ischemia, hypoxia, and necrosis (1). MDRPI is one of the common complications in surgical patients, especially in prolonged surgeries (2). PI not only increase patient pain, prolong hospitalization and increase healthcare costs (3–5), but also may lead to serious complications such as infection and sepsis, which can endanger the patient’s life (4). Respiratory devices, including endotracheal tubes, often have MDRPIs (2, 6, 7). The technique of transnasal tracheal intubation is widely used in oral cavity, head and neck surgery because it provides the surgeon with a better view of the operation (8).

As a specialized dental hospital, which is common procedure used the transnasal tracheal intubation technique. During prolonged procedures, the transnasal tracheal tube exerts continuous pressure on the nasal skin and mucosa, which in turn causes ischemic and hypoxic damage to the nasal tissues and predisposes to transnasal tracheal tube-related nasal PI (8, 9). In recent years, the incidence of malignant tumors of the oral cavity, head and neck has continued to rise, and related surgeries have increased year after year, with prolonged surgical times and an increased incidence of nasal PI (8, 9). Nasal PI not only affects the patient’s facial aesthetics that cannot be easily repaired (10) and increases the patient’s pain and treatment costs, but may also prolong hospitalization and affect the patient’s recovery and prognosis. Routine nursing measures, such as the use of hydrocolloid dressings or modified methods of tracheal tube fixation, although effective in preventing nasal PI (8, 11–13), still have limitations in prolonged surgery. Furthermore, the incidence of PI is of concern to all levels of healthcare as a sensitive indicator of quality of care (14). Therefore, hospitals pay special attention to the important topic of preventing the occurrence of nasal PI.

In recent years, the Multidisciplinary Collaboration Model (MCM) has not only gained widespread use and recognition in the medical field, but also plays an important role in the prevention of stress injuries (11). By integrating the expertise and skills of different disciplines, it is possible to assess risk more comprehensively and develop targeted interventions. Under the auspices of the hospital’s Patient Safety Initiative, in 2024 we undertook a special improvement activity for the prevention of intraoperative transnasal tracheal tube-related nasal pressure injuries in a multidisciplinary collaborative model.

## Materials and methods

This is a single-center, historical controlled study to investigate the clinical effectiveness of implementing a multidisciplinary collaborative model for the prevention of transnasal airway catheter-related nasal PI in patients undergoing prolonged oral, head and neck surgery as a special quality improvement activity in a municipal dental specialty hospital in China. The historical control group, which received routine preventive measures, was compared with the intervention group, which received a multidisciplinary, collaborative, whole process prevention and control strategy. We hypothesized that effective prevention of nasal PIs in patients undergoing prolonged surgery and transnasal endotracheal intubation requires the implementation of a multidisciplinary collaborative model and improvement of prevention strategies.

This special improvement activity was carried out in 2024 at the Affilated Stomatological Hospital of Chongqing Medical University, People’s Republic of China. This hospital is a major municipal hospital specialized in oral and maxillofacial surgery. The operating room is the main site of the study.

The sample for this study was determined from the chart review based on the inclusion exclusion criteria. The sample was selected from surgical patients who underwent elective oral, head and neck surgeries between January 2023 and December 2024 at the hospital.

The historical control group was identified through chart review; between January and December 2023, the hospital used routine care measures for nasal PI prevention and control, and 221 surgical patients who met the inclusion and exclusion criteria were selected as a sample. The intervention group was identified as 294 surgical patients who met the inclusion exclusion criteria as a sample of 294 surgical patients who met the inclusion exclusion criteria during the period of January to December 2024, when the hospital implemented a total nasal PI prevention and control strategy in a multidisciplinary collaborative model.

This study was a retrospective analysis designed to assess the effectiveness of implementing quality improvement activities to reduce the incidence of nasal PI in 2024. In 2023, 221 patients were enrolled, of whom 16 had nasal PI (7.24% incidence); in 2024, 294 patients were enrolled, of whom 7 had nasal PI (2.38% incidence), for a total sample size of 515. The sample size was calculated for the comparison of two independent proportions using a two-sided significance level of 0.05 and a target power of 80%. Based on historical quality surveillance data from 2023, the incidence of nasal pressure injury was estimated at 7.24%. For this quality improvement initiative, a clinically meaningful and achievable reduction to approximately 2.0% was prespecified. Allowing for unequal group sizes consistent with the study periods, the minimum required sample size was approximately 194 patients in the control group and 257 in the intervention group. The final sample (221 vs. 294; total 515) exceeded this requirement.

Inclusion criteria for the historical control group and the post-implementation intervention group: ① surgery time ≥3 h; ② age ≥18 years; ③ BMI of 18.5–23.9 kg/m^2^; ④ transnasal tracheal intubation. Exclusion criteria for the historical control and post-implementation intervention groups: ① preoperative comorbid diabetes mellitus or severe malnutrition or uncontrolled hypertension; ② preoperative nasal skin or mucous membrane injury, infection, or history of previous nasal surgery; ③ preoperative coagulation dysfunction or use of therapeutic doses of anticoagulant medications; and ④ emergency surgery or loss of visitation within 24 h of the operation, which prevented nasal evaluation.

The multidisciplinary collaborative nasal PI prevention and control strategy was developed in January 2024 with the support of the hospital’s Patient Safety Initiative. The multidisciplinary team consists of medical administrators, nursing administrative personnel, anesthesiologists, surgeons, wound therapists, and operating room nurses. The team members receive PI-specific training (15) to ensure that each member has the relevant knowledge and skills, and then work together to implement a full process prevention and control strategy that consists of a structured, comprehensive, and dynamic risk assessment—a dynamic graded intervention—and a structured post-operative follow-up visit.

Medical administrators and nursing administrative personnel work together to develop prevention and control strategies and monitor the quality of implementation. Wound therapists perform PI risk assessments preoperatively and postoperatively using the Braden Risk Assessment Form, and structured return visits were performed within 24 h postoperatively for nasal skin status. Operating room nurses performed dynamic PI risk assessments and implemented graded interventions using the modified Competency, Outcomes, and Resources in Nursing (CORN) scale to assess the risk of nasal pressure injuries preoperatively, intraoperatively, and postoperatively.

Nasal skin assessments were conducted immediately after extubation and at 24 h postoperatively. While most acute nasal injuries are visible within this timeframe, it is noted that this follow-up period primarily focuses on the immediate impact of the medical device during the perioperative phase.

Anesthesiologists, surgeons, and operating room nurses dynamically adjusted the graded interventions according to the risk level, including: selecting an appropriate thickness of tracheal tube and lubricating it adequately before anesthesia, applying eye ointment or petroleum jelly or Sai Botanic Lotion to the skin in contact with the nose of the nasotracheal tube (3, 12, 16); wrapping the nasotracheal tube, the filter, and the respiratory tubing and their connections with a disposable transparent and aseptic protective sleeve before surgery; maintaining optimal suture tension—secure enough to prevent tube dislodgment but sufficiently loose to avoid mechanical tissue compression during surgery; and removing the sutures in time after the operation. Paying attention to the skin of the nose during the operation, and adjusting the position of the transnasal endotracheal tube visually in real time so that the endotracheal tube hangs as far as possible in the nasal cavity. The “mobile nursing system” functioned as a decision-support tool, automatically triggered prompts based on real-time CORN scores:

1. Preoperative nursing interventions: low risk: routine lubrication and standard fixation. Medium risk: use of eye ointment/petroleum jelly/Sai Botanic Lotion at contact points; wrap nasotracheal tube (NTT), filters, etc., with laparoscopic camera sleeve.
2. Intraoperative nursing interventions: low risk: monitor nasal skin condition. Medium risk: remind surgeon about appropriate suture tension for nasal/NTT fixation; adjust NTT position; remind surgeon not to suture-fix NG tube to NTT and to remove promptly post-op; keep NTT suspended in nasal cavity as much as possible. High risk: all medium-risk measures plus apply eye ointment, moisturizer, or povidone-iodine to nasal area during wound closure.

The multidisciplinary bundle and the specific intervention algorithms according to different risk levels are summarized in Table 1.

**Table 1:** Multidisciplinary bundle and graded intervention algorithm for nasal PI prevention.

Stage	Risk level (CORN score)	Intervention components	Responsible clinician	Frequency/trigger
Preoperative	Low risk (<9)	Routine lubrication of the nasotracheal tube (NTT). Standard NTT fixation.	Anesthesiologist, OR Nurse	Before induction
	Medium/high risk (≥9)	Apply eye ointment, petroleum jelly, or Sai Botanic Lotion to nasal contact points. Wrap NTT, filters, and respiratory tubing with a disposable transparent sterile protective sleeve.	Anesthesiologist, OR Nurse	Immediately after intubation
Intraoperative	All levels	Dynamic monitoring of nasal skin condition and vital signs.	OR Nurse	Every 2 hours
	Medium risk (8–12)	Adjust NTT position via visual guidance through the transparent sleeve. Keep NTT “suspended” (airborne) in the nasal cavity as much as possible. Remind surgeon to maintain optimal suture tension and avoid fixing the NG tube to the NTT.	OR Nurse, Surgeon	Triggered by “Medium Risk” score in mobile system
	High risk (>12)	Implement all Medium Risk measures. Re-apply eye ointment or moisturizer or povidone-iodine to the nasal area during wound closure.	OR Nurse, Anesthesiologist, Surgeon	Continuous monitoring until surgery ends
Postoperative	All levels	Prompt removal of fixation sutures after surgery. Structured follow-up of nasal skin status within 24 h.	Surgeon, Wound Therapist, OR Nurse,	Upon extubation and 24 h post-op

The disposable transparent sterile protective sleeve (material: transparent polyethylene; dimensions: standard sterile camera sleeve 15 cm × 200 cm; manufacturer: Xuchang Zhengde Medical Products Co.) was used (Supplementary Figure 1). This EO-sterilized sleeve was applied to wrap the transnasal endotracheal tube, filter, and respiratory tubing, allowing for a clear view of the nasal pressure points while maintaining a sterile barrier (Supplementary Figure 2). Its low weight (approx. 24 g) significantly reduced the external load compared to traditional sterile towels.

The key to this quality improvement activity is multidisciplinary collaboration and division of labor to complete assessments and implement precise interventions. Therefore, prior to the start of the improvement activity, a one-week special training was conducted for team members, with each member participating in at least two training sessions and simulation exercises, and an assessment was conducted to ensure that 100% of the team members mastered the use of the assessment forms and the correct implementation of the interventions. The multidisciplinary team utilized a modified CORN scale for dynamic risk assessment (see Supplementary Material S1 for the full scale). To ensure inter-rater reliability, all assessors underwent simulation training, achieving a Kappa coefficient of 0.85 before the study began. Postoperative assessments of nasal skin status within 24 h were conducted by wound therapists who were not part of the intraoperative care team. These assessments followed the standard staging criteria (NPUAP) with all diagnosed cases cross-checked by a second independent senior nurse to reduce observer bias.

The protocol for this study was approved by the Ethics Committee of The Affiliated Stomatological Hospital of Chongqing Medical University (no. 2025 Clinical Review [037]). Since this study was a retrospective analysis of clinical data collected during a quality improvement project (2023–2024), the Ethics Committee granted retrospective approval.

### Statistical analysis

The relevant data for 2023 and 2024 were extracted into Excel spreadsheets through the hospital’s Madison surgical anesthesia system, and mobile care system, respectively. The principal investigator reviewed the medical record data based on inclusion and exclusion criteria.

Surgical patients were compared one year before and after the implementation of this quality improvement activity. The incidence of nasal PI was the outcome metric of the study. This indicator directly reflects the effectiveness of the intervention and facilitates comparison of the effectiveness of the intervention between the two groups, the historical control group (before the quality improvement activity was implemented) and the intervention group (after the quality improvement activity was implemented). Data for both groups were extracted through chart review.

Basic patient data, including age, gender, height, and weight, were collected through the Madison Anesthesia Clinical Information System, as well as surgery-related data, including type of surgery, anesthesia modality, anesthesia start time, and surgery end time. Data related to the patient’s health status were collected through the mobile nursing system, including the presence of preoperative comorbidities of diabetes mellitus, severe malnutrition, and uncontrolled hypertension; preoperative nasal skin or mucosal injuries, infections, and history of previous nasal surgery; preoperative coagulation dysfunction and use of therapeutic doses of anticoagulant medications; and the occurrence of intraoperative nasal PI.

To minimize the inherent bias associated with a historical control design, we ensured that the clinical environment remained consistent throughout the study period (2023–2024). Specifically, there were no significant changes in the composition of the anesthesia teams, the nurse-to-patient staffing ratios in the operating rooms, or the standard intraoperative temperature management protocols. All surgical procedures were performed using identical models of nasotracheal tube and medical equipment across both groups. Furthermore, the postoperative care pathways followed a unified standard operating procedure (SOP), ensuring that the observed differences in nasal pressure injury (PI) rates were primarily attributable to the multidisciplinary intervention rather than external co-interventions or secular trends.

All statistical analyses were performed using SPSS version 21.0 (IBM Corp., Armonk, NY, United States). Continuous variables were assessed for normality using the Kolmogorov–Smirnov test and are presented as mean ± standard deviation (x̅ ± s). Between-group comparisons of continuous variables were conducted using independent-samples t tests. Categorical variables are presented as counts and percentages and were compared using the chi-square test or Fisher’s exact test, as appropriate.

The primary outcome was the incidence of nasal pressure injury. Differences in incidence between the historical control group and the intervention group were evaluated using a two-sided test with a significance level of *α* = 0.05. Effect sizes were reported as absolute risk reduction (ARR), relative risk (RR), and number needed to treat (NNT), each with corresponding 95% confidence intervals (CIs). ARR and its 95% CI were calculated using the Newcombe method for two independent proportions. NNT and its 95% CI were derived from the inverse of the ARR and its confidence limits. Relative risk and 95% CI were estimated using the log-transformed method.

All statistical tests were two-sided, and a *P*–value < 0.05 was considered statistically significant.

## Results

A total of 370 patients were assessed for eligibility between January and December 2023. Of these, 149 were excluded. A total of 467 patients were assessed for eligibility between January and December 2024. Of these, 173 were excluded. The patient screening, inclusion, and exclusion process is summarized in the flow diagram (Supplementary Figure 3).

**Figure 1:**
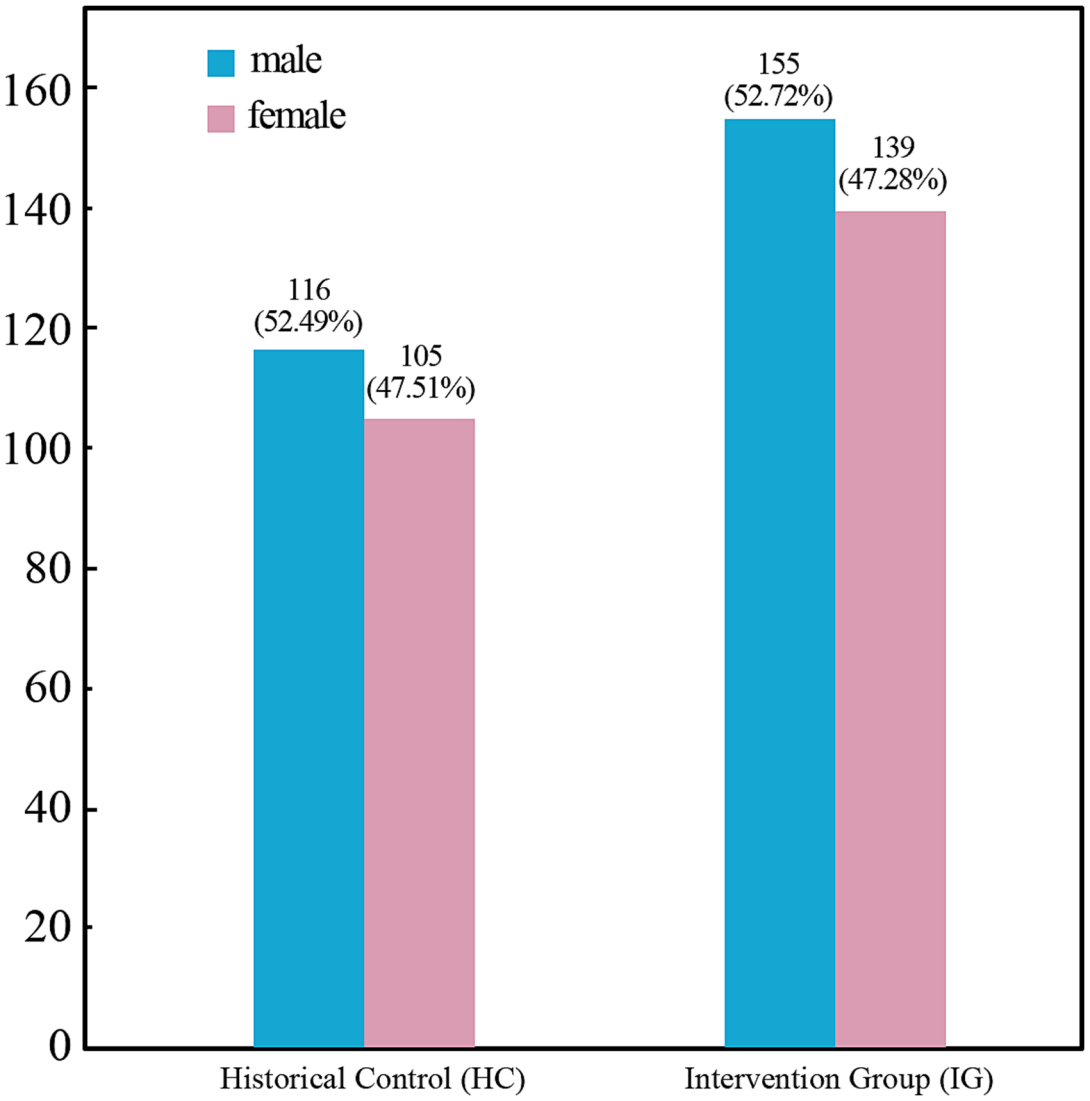
Comparison of gender distribution between the historical control (HC) and intervention group (IG). Comparison of ender distribution between HC and IG cohorts. The bar charts display the gender ratio, showing no significant difference between the historical control group (*n* = 221) and the intervention group (*n* = 294) (*p* > 0.05), ensuring baseline comparability.

**Figure 2:**
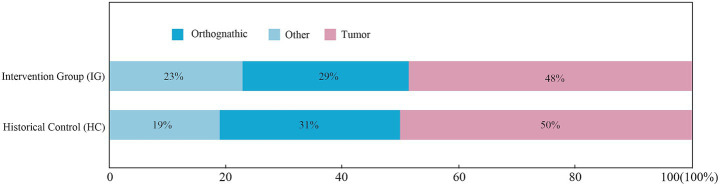
Distribution of surgery types in historical control (HC) and intervention group (IG) patients. Distribution of surgical types in study populations. The stacked bar charts compare the proportions of tumor, orthognathic, and other oral/head/neck surgeries. Statistical analysis confirmed a balanced distribution across surgical categories (*p* > 0.05), minimizing confounding related to surgical complexity.

The study sample consisted of 515 adult surgical patients who met inclusion exclusion criteria between 2023 and 2024, including a historical control group (*n* = 221) and an intervention group after implementation of quality improvement activities (*n* = 294). Comparison of gender and type of surgery between the two groups is shown in Figures 1, 2, respectively, and the differences were not statistically significant (*p* > 0.05). As summarized in Table 2, the baseline clinical characteristics, including age, body mass index (BMI), and duration of surgery, were well-balanced between the historical control (HC) and intervention groups (IG). There were no statistically significant differences observed across all parameters (all *p* > 0.05). Furthermore, the Standardized Mean Differences (SMD) for all baseline variables were below the threshold of 0.1, indicating that the two cohorts were highly comparable. This high degree of baseline balance minimizes the potential impact of confounding factors on the primary outcome of nasal pressure injuries.

- Continuous variables (Age, BMI, and Duration of surgery) are expressed as mean ± standard deviation (x̅ ± s).
- Inter-group comparisons were performed using the independent-samples *t*-test; all tests were two-sided, with *p* < 0.05 considered statistically significant.
- SMD < 0.1 was pre-specified as the threshold to indicate a negligible difference between the two cohorts, ensuring that the historical control and intervention groups were well-balanced and comparable, thereby minimizing potential confounding bias.

**Table 2:** Comparison of baseline characteristics between HC and IG groups.

Characteristic	HC (*n* = 221)	IG (*n* = 294)	Statistic (*t*)	*p*-value	SMD
Age (years)	46.53 ± 19.12	47.54 ± 20.23	−0.573	0.567	0.051
BMI (kg/m^2^)	21.19 ± 1.48	21.11 ± 1.61	0.576	0.565	0.052
Duration of surgery (h)	6.17 ± 2.35	6.32 ± 2.46	−0.710	0.478	0.063

HC, Historical Control group; IG, Intervention Group; BMI, Body Mass Index; SMD, Standardized Mean Difference.

Table 3 summarizes the clinical outcomes of the historical control group and the intervention group after the implementation of quality improvement activities. Sixteen nasal PIs occurred in the historical control group, of which 13 were stage 1 and 3 were stage 2; seven nasal PIs occurred in the intervention group, all of which were stage 1, and none of which were stage 2; no nasal PIs of stage 3 or higher occurred in either group. The incidence of nasal pressure injury decreased from 7.24% (16/221) in the historical control group to 2.38% (7/294) in the intervention group. The absolute risk reduction was 4.86% (95% CI 1.19–9.23%), corresponding to a number needed to treat of 21 (95% CI 11–85). The intervention was associated with a significantly lower risk of nasal pressure injury (RR 0.33, 95% CI 0.14–0.79). The between-group difference remained statistically significant using a two-sided test (Z = 2.64, two-sided *p* = 0.008) (Table 3). Notably, no ventilation safety incidents, accidental line disconnections, or respiratory complications occurred during the intervention period, confirming the safety of the visual adjustment protocol.

- Categorical data (Nasal PI) are expressed as numbers and percentages [*n* (%)].
- Inter-group comparison was performed using a two-sided Z-test; *p* < 0.05 was considered statistically significant.
- ARR represents the absolute difference in PI rates between groups; RR represents the ratio of PI risk in the IG versus the HC group; NNT represents the number of patients who need to receive the intervention to prevent one additional nasal PI.
- The 95% CI for effect size measures indicates the precision of the estimated treatment effect.

**Table 3:** Clinical outcomes and effect size measures of nasal PI in HC and IG patients.

Outcome	HC (*n* = 221)	IG (*n* = 294)	Statistic (*Z*)	*P*-value (two-sided)
Nasal PI, *n* (%)	16 (7.24%)	7 (2.38%)	2.64	0.008

Effect size measures	Value	95% CI
ARR (%)	4.86	1.19–9.23
RR	0.33	0.14–0.79
NNT	21	11–85

HC, Historical Control group; IG, Intervention Group; PI, Pressure Injury; ARR, Absolute Risk Reduction; RR, Relative Risk; NNT, Number Needed to Treat; CI, Confidence Interval.

## Discussion

Nasal pressure injury is one of the common complications in patients undergoing transnasal endotracheal intubation, which is mainly due to local tissue ischemia and hypoxic injury caused by continuous direct pressure (12) exerted on the nasal skin and mucosa by the transnasal endotracheal tube and its attached filters and respiratory threaded tubing. Although routine nursing measures, such as nasal application of hydrocolloid dressings or modified methods of tracheal tube immobilization (12, 13, 17) are effective in preventing nasal PI, they have significant limitations in dealing with the complex scenarios of prolonged surgery. These routine care measures make it difficult to achieve dynamic redistribution of pressure, much less effectively reconcile the conflicting demands of anesthetic operations (e.g., catheter fixation stability and ventilation safety) with the demands of surgical procedures (consistently optimized exposure of the operative field). Therefore, for such high-risk surgical patients, there is an urgent need for a systematic prevention and control strategy that goes beyond a single nursing perspective, and a systematic prevention and control system can only be constructed through the integration of anesthesia, nursing, surgery, and wound management and the adoption of a multidisciplinary collaboration model.

In this study, a special quality improvement program relying on a multidisciplinary collaborative model was successfully implemented with the support of a special patient safety initiative in a Chinese municipal dental specialty hospital. At the heart of the project is the formation of a collaborative team that integrates the professional strengths of medical administration, nursing administration, anesthesiology, surgery, wound care, and operating room nursing. Through the implementation of the whole-process prevention and control strategy of “structured and comprehensive dynamic risk assessment—dynamic graded nursing intervention—postoperative structured return visit”, the incidence of nasal pressure injuries associated with nasotracheal tube was significantly reduced from 7.24% in the historical control group to 2.38% in the intervention group, and the occurrence of serious injuries of stage 2 and above was effectively eliminated. This remarkable result strongly confirms the superiority of the multidisciplinary collaboration model in solving complex clinical problems and improving patient safety and quality.

The successful implementation of this project relies on the synergy of a number of key strategies, two of which stand out as constituting the core innovation of the study:

1. The most groundbreaking innovation of this study is the preoperative use of a disposable transparent sterile protective sleeve to completely wrap the transnasal endotracheal tube, filter, and respiratory threaded tubing and their connection sites. Compared with the improvement of using 2 sterile cloth towels for wrapping, this technique offers three significant advantages: on the one hand, the weight of the wrapping material exerts direct pressure on the nose through the transnasal airway tube, and compared with 2 sterile cloth towels (approximately 175-250 g), a single disposable clear protective sleeve weighs approximately 24 g, which significantly reduces direct pressure on the nose through the transnasal airway tube over a prolonged period of time. On the other hand, we focus on the regular readjustment of the position of the transnasal airway tube. The disposable transparent sterile protective cover provides an unobstructed and clear view, which enables the operating room nurses to directly and real-time observe the specific pressure point of the transnasal airway tube on the nasal tissues during the operation, and combined with the results of the dynamic risk assessment, the team was able to timely and accurately adjust the position of the transnasal airway tube on the nose, and to achieve the dynamic redistribution of pressure (18), which fundamentally improves the disadvantages of the traditional fixation, which is difficult to adjust or requires “blind” adjustment. Furthermore, while optimizing the pressure adjustment, the disposable transparent sterile protective sleeve tightly ensures the sterile barrier and physical tightness of the respiratory line connection, completely eliminating the risk of accidental disconnection or contamination of the line that may be triggered by intraoperative adjustment operations, thus fundamentally safeguarding the patient’s ventilation safety. This innovation cleverly resolves the tension between the need for pressure redistribution and the safety and security of breathing lines, which is often difficult to reconcile in conventional methods.
2. The supporting core strategy is the construction of a dynamic assessment and graded intervention system that is structured and managed throughout the process. The system employs a dual system of risk assessment: an overall pressure injury (PI) risk assessment of the patient by the wound therapist using the Braden Risk Assessment Scale preoperatively and postoperatively; meanwhile, the OR nurse performs a dynamic, specialized risk assessment using the modified CORN Intraoperative Acquired PI Risk Assessment Scale preoperatively, intraoperatively, and postoperatively. The key innovation of the Modified CORN Intraoperative Acquired PI Risk Assessment Scale is the embedded specifics of graded interventions that correspond to the risk level assessed in real time. The mobile nursing system automatically triggers and prompts graded interventions corresponding to the corresponding risk level based on the risk level assessed in real time, which greatly enhances the timeliness and accuracy of the team’s response and ensures that medium- and high-risk patients are provided with more timely and accurate preventive measures, an innovation that further realizes information technology-driven precision interventions. In terms of interventions, a multi-component “combo” has been developed: At the source control level, the anesthesiologist selects a catheter of appropriate thickness based on the patient’s anatomy and lubricates it adequately, and preapplies eye ointment, petroleum jelly, or Sai Botanical Lubricant to reduce friction at the site where the catheter comes into contact with the nose (3, 12, 16), and then uses the same type of fixation tape and the same type of fixation; During the intraoperative dynamic maintenance session, the surgeon is responsible for fine-tuning the tightness of the nasal fixation sutures and ensuring that they are removed in a timely manner, while the operating room nurse performs the aforementioned visual catheter adjustments and makes every effort to ensure that the endotracheal tube remains as “airborne” as possible within the nasal cavity in order to minimize direct pressure on the nasal tissues. Finally, a structured post-operative visit within 24 h to assess the condition of the nasal skin creates a closed loop of “assessment-intervention-feedback” to ensure early identification and management of any potential problems.

The key to the success of the multidisciplinary collaboration model is the establishment of a clear division of specialization and an efficient synergy mechanism:

1. Leadership and quality control. In this quality improvement activity, experts from the Department of Medical Affairs and the Department of Nursing worked together to develop a framework of prevention and control strategies and quality control standards based on evidence-based rationale (18, 19), organized regular quality analysis meetings, monitored the implementation process and promoted continuous improvement, and provided key organizational safeguards and professional guidance, which were critical to the success of the project (20).
2. Professional technical implementation, including anesthesiologists leading the selection of transnasal tracheal catheter model (18), lubrication and provide positional adjustment of professional advice and security; maxillofacial surgeons accurately regulate the tightness of the patient’s nasal fixed sutures, timely removal of sutures; The wound therapist conducts PI risk assessment, provides professional guidance on skin protection, leads the development and application of PI science videos and picture books for preoperative education, and enhances the knowledge of doctors and patients about PI; The operating room nurse, as the implementer and coordinator, is responsible for performing dynamic preoperative, intraoperative, and postoperative assessments using the modified CORN Intraoperative Acquired Pressure Injury Risk Assessment Scale, implementing graded interventions, performing visual catheter adjustments, monitoring nasal conditions in real time, and performing structured return visits within 24 h of surgery to ensure that potential problems are detected and addressed early.
3. The multidisciplinary collaboration model breaks down disciplinary barriers and promotes information sharing and resource integration. A week-long special training and simulation exercise ensured that 100% of the team members mastered the assessment tools and intervention process. Regular interdisciplinary exchange meetings and trainings have effectively brokendown professional barriers, facilitated the sharing of expertise and integration of experience, and developed and standardized actionable operational standards for nasal PI, which not only enhances the relevance and effectiveness of interventions, but also reduces duplication of efforts and waste of resources.

The results of this study suggest that a dedicated quality improvement campaign in a multidisciplinary collaborative model with an integrated strategy of combining multiple preventive measures significantly reduces the incidence of nasal PI associated with transnasal tracheal catheters. This finding is highly consistent with existing relevant international literature emphasizing the effectiveness of multidisciplinary collaboration and comprehensive prevention strategies. For example, studies by Guido Ciprandi et al. (21), Delmore et al. (22), and Pittman et al. (18) point to the fact that the proper application of comprehensive prevention strategies by interdisciplinary teams can be effective in preventing the occurrence of stress injuries. Studies by Gupta et al. (3), Chaboyer et al. (23), and Gaspar et al. (24) have also demonstrated that multidisciplinary teams can significantly reduce the incidence of stress injuries through PI risk assessment and proper implementation of a range of preventive measures. The unique value and contribution of this study is:

1. Technological innovation. For the first time, we have proposed and practiced the use of disposable transparent sterile protective sleeve for visual catheter adjustment technology, which provides an innovative solution for realizing dynamic redistribution of pressure during operation and guaranteeing the safety of respiratory line, especially suitable for oral head and neck surgery with high requirements for both the operation field and the safety of ventilation.
2. Specialized hospital application verification. For the first time, the effectiveness of the multidisciplinary collaborative whole-process prevention and control model for nasal PI associated with transnasal tracheal tube has been systematically constructed and validated in the specific scenario of a municipal dental specialty hospital in China, providing a practical template that can be borrowed by similar specialty institutions.
3. Structured process and information technology empowerment. A clear structured assessment-intervention-monitoring-return-visit process has been established, and through the combination of the improved CORN intraoperative acquired stress injury assessment scale with embedded intervention prompts and the mobile nursing system, a rapid and precise translation of risk assessment results to interventions has been realized, enhancing process efficiency.
4. Patient experience enhancement. Patients spoke highly of the systematic and refined care under the multidisciplinary collaboration model, believing that it not only improved the quality of care, but also enhanced their sense of security and trust in medical services, helping to improve the doctor-patient relationship and enhance the hospital’s reputation.

Despite the positive outcomes, several limitations should be acknowledged. First, this study utilized a single-center, historical controlled design, which is inherently susceptible to secular trends and temporal confounding. While we have adjusted for key factors such as age, BMI, and surgery duration, other variables like subtle shifts in surgical case-mix or anesthesia staffing over time might influence the outcomes. However, given that our department maintained stable staffing and standardized perioperative protocols during the study timeframe, the impact of these variables is likely minimal. Future prospective randomized controlled trials are needed to further validate these findings. Second, the follow-up duration for nasal pressure injuries was limited to 24 h post-operation. Existing literature suggests that some medical device-related pressure injuries may fully manifest within 48 to 72 h. Therefore, our study may have underestimated the true cumulative incidence of late-evolving injuries. However, given that the majority of transnasal tubes in this study were removed shortly after surgery, the 24-h assessment captured the peak period of direct device-related stress. Future research should implement longer monitoring windows to capture the full progression of these injuries. Third, the modified CORN scale, while integrated into our mobile nursing system for real-time prompts, was used as a site-specific assessment tool for nasal injuries. Its specificity and predictive validity for this particular anatomical site require further validation and optimization through prospective studies with larger, multi-center cohorts. Finally, as this quality improvement project was conducted in a specialized dental hospital focusing on oral, head, and neck surgeries, the findings may not be directly generalizable to other surgical specialties or different clinical environments. Future research should employ multicenter, prospective, or stepped-wedge cluster randomized designs to confirm the long-term clinical and cost-effectiveness of this multidisciplinary collaborative model.

## Conclusion

This study amply demonstrates that a multidisciplinary collaborative model of specialized quality improvement activities for the prevention of nasal pressure injuries associated with transnasal tracheal catheters in a specialized municipal dental hospital. By breaking down professional barriers, integrating technical resources, and constructing a closed-loop management system of “assessment-intervention-monitoring-return visit,” the incidence of nasal PI associated with transnasal tracheal catheters in patients undergoing prolonged surgery of the oral cavity, head and neck can be significantly and effectively reduced, and the occurrence of serious injuries of Stage 2 and above can be effectively prevented. This successful practice not only significantly improves quality of care sensitivity indicators, enhances patient safety and experience, provides new ideas and methods for nasal PI prevention, but also provides a replicable and scalable systematic solution for the prevention of such complications in similar specialized medical institutions. Future research should focus on validating the generalizability of key technologies, optimizing risk assessment tools, evaluating long-term benefits and cost-effectiveness, and exploring the expansion of this multidisciplinary collaborative model to other areas of prevention of high-risk medical device-related PIs.

## Data Availability

The original contributions presented in the study are included in the article/Supplementary material, further inquiries can be directed to the corresponding author.
