# Supplementary material for: Clinical effectiveness of a multidisciplinary quality improvement initiative to prevent nasal pressure injuries associated with nasotracheal tube: a historical controlled study
Source: Front Med (Lausanne). 2026 Apr 8;13:1744744. doi: 10.3389/fmed.2026.1744744 (PMC13101432; doi:10.3389/fmed.2026.1744744)
Supplement: Supplementary file 1 [file Supplementary_file_1.docx]

Supplementary Figure 1: Material and Specifications of the Transparent Protective Sleeve


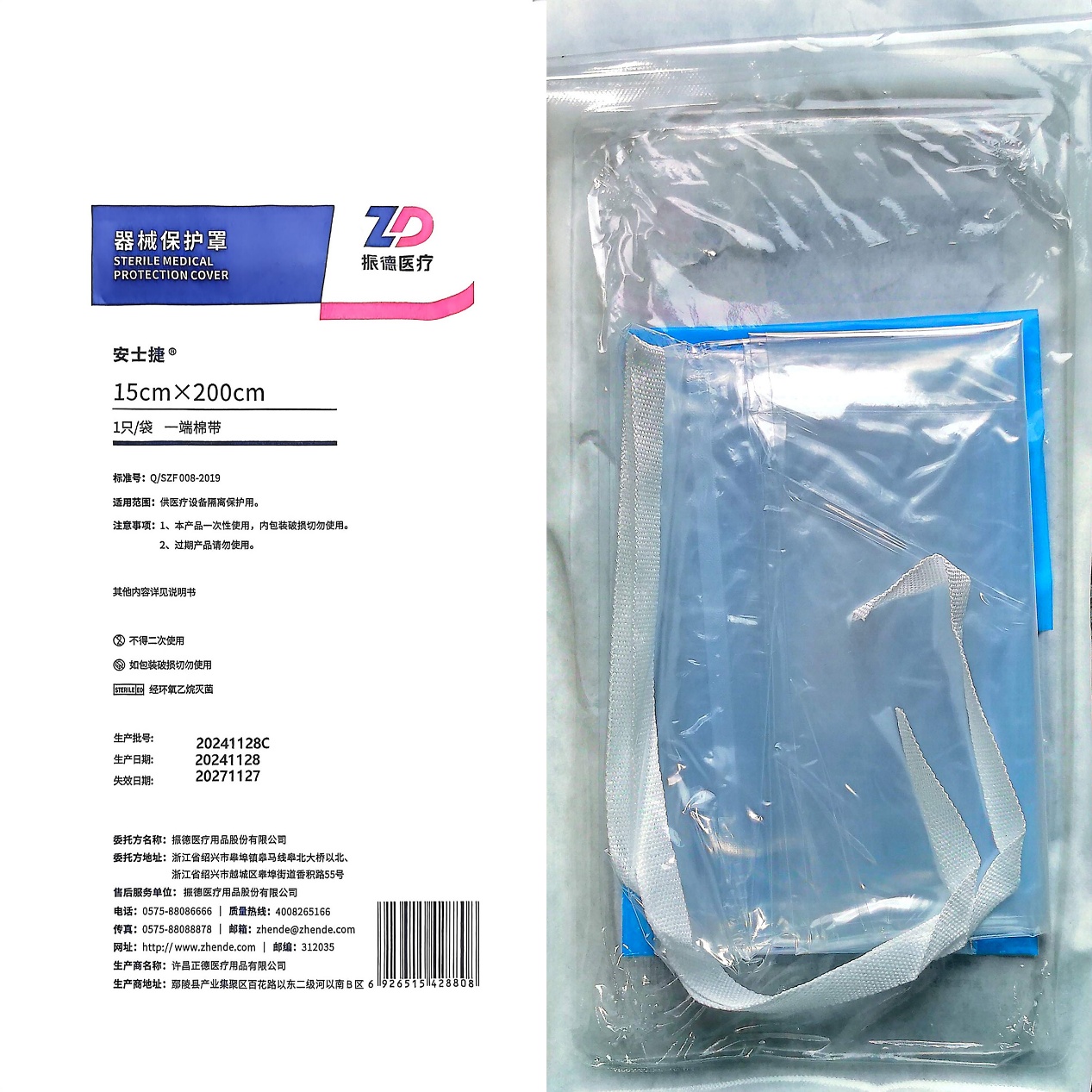


Supplementary Figure 1. Material and Specifications of the Transparent Protective Sleeve. The sleeve is made of medical-grade transparent polyethylene with dimensions of 15 cm × 200 cm. This EO-sterilized device is designed to wrap the tracheal tube and breathing circuits, providing a sterile barrier while maintaining high visibility for pressure point monitoring.

Supplementary Figure 2: Clinical Application and Installation of the Transparent Protective Sleeve

**
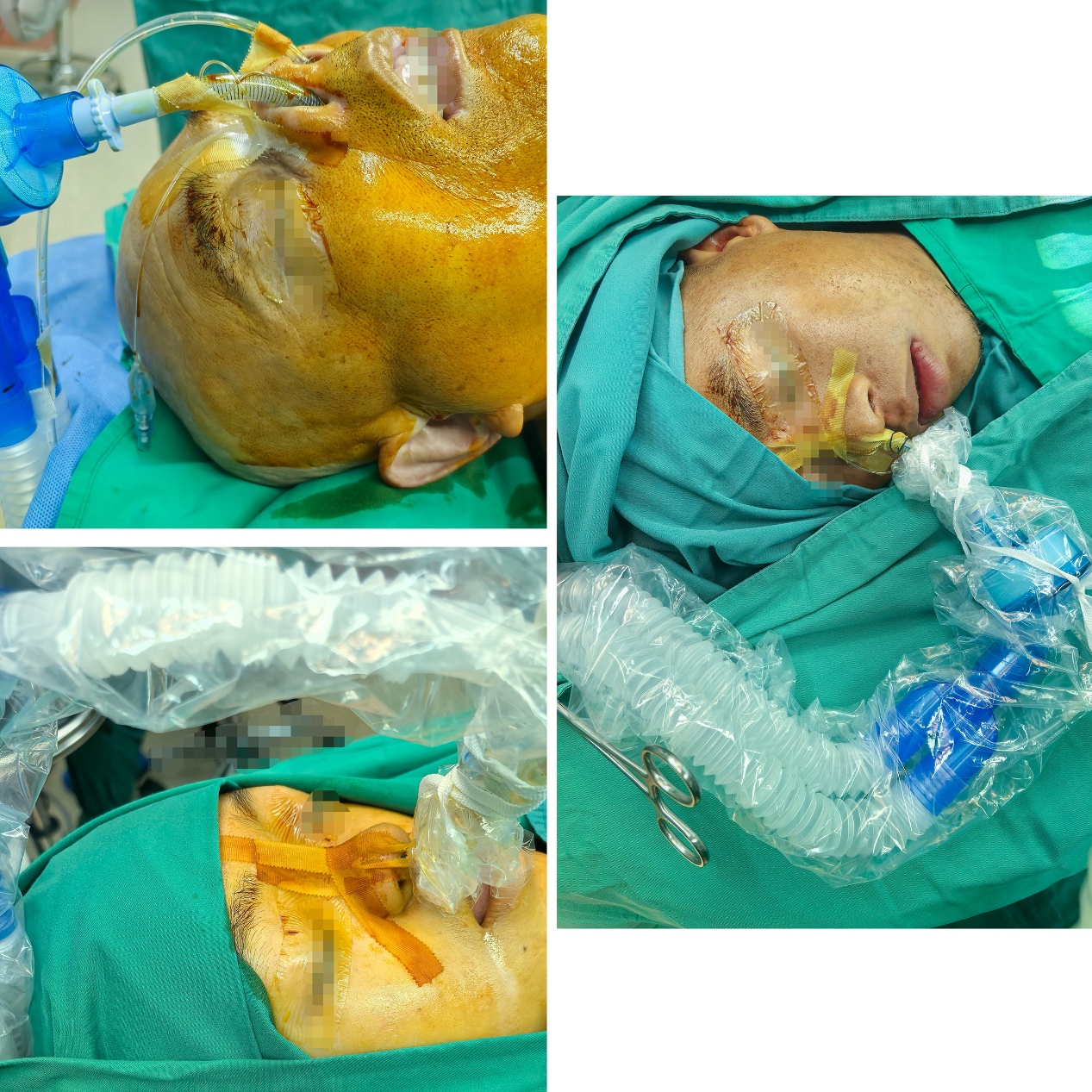
**

Supplementary Figure 2. Clinical Application and Installation of the Transparent Protective Sleeve. The diagram illustrates the application of the lightweight (approx. 24 g) sleeve. By wrapping the nasotracheal tube and filters, it allows operating room nurses to visually monitor nasal pressure points in real-time and facilitate dynamic tube adjustment without breaking the sterile field.

Supplementary Figure 3: Flowchart of Patient Screening and Enrollment based on SQUIRE/STROBE Guidelines

**
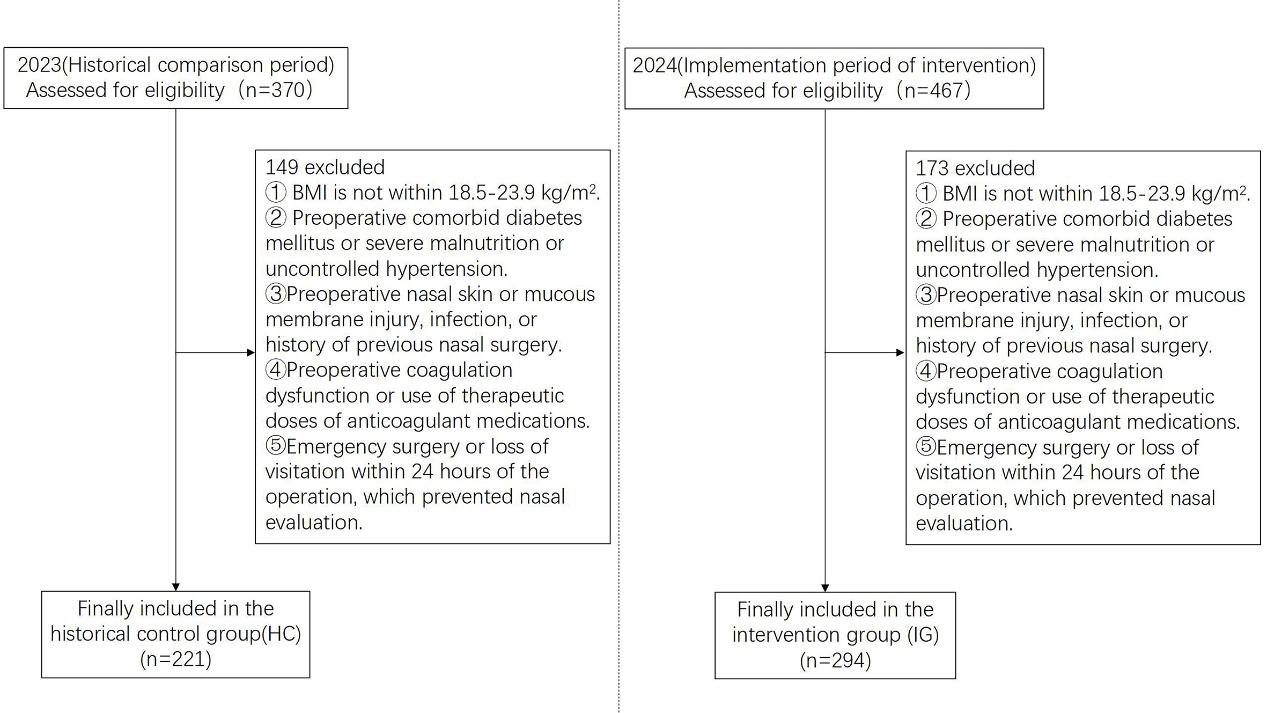
**

Supplementary Figure 3. Flowchart of Patient Screening and Enrollment based on SQUIRE/STROBE Guidelines. The diagram details the progression of participants from initial screening to final group allocation. A total of 837 patients were assessed, with 322 excluded based on abnormal BMI or underlying medical conditions.
