# Supplementary material for: Clinical effectiveness of a multidisciplinary quality improvement initiative to prevent nasal pressure injuries associated with nasotracheal tube: a historical controlled study
Source: Front Med (Lausanne). 2026 Apr 8;13:1744744. doi: 10.3389/fmed.2026.1744744 (PMC13101432; doi:10.3389/fmed.2026.1744744)
Supplement: Supplementary file 2 [file Data_Sheet_1.pdf]

CORN Intraoperative-Acquired Pressure Injury Risk Assessment Scale (Revised Version)

Department:　Bed No.:　Name:　Gender:　Age:　Hospitalization No.:　Surgery Date:

Preoperative Pressure Injury Risk Factor Assessment (Mark √ in ☐, Total Score: ______ points, ______ Risk)

Item & Assessment	1 point	2 point	3 point	4 point
ASA Physical Status	Class I ☐	Class II ☐	Class III ☐	≧Class IV ☐
Body Mass Index (BMI)	18.5-23.9 ☐	24.0-27.9 ☐	≧28 ☐	<18.5 ☐
Skin Condition at Pressure Sites	Intact ☐	Erythema, Moisture ☐	Bruising, Blister ☐	Severe Edema ☐
Preoperative Limb Mobility	Unrestricted ☐	Mildly Limited ☐	Partially Limited ☐	Completely Limited ☐
Estimated Surgery Time (h)	<3 ☐	≧3 &<3.5 ☐	≧3.5 &<4 ☐	≧4 ☐
High-Risk Disease (Diabetes)				Yes ☐

Preoperative Assessment:> 14 points = High-Risk; 9 - 14 points = Medium-Risk; <9 points = Low-Risk.

Nursing Interventions:

Low Risk: ☐Standard positioning on operating table ☐Use memory foam OR mattress ☐Heel suspension
☐Keep skin, sheets clean, dry, flat ☐Ensure medical devices are appropriately tight
☐Use dressings/padding at skin-device/instrument interface
☐Use appropriately sized and lubricated endotracheal tube (ETT)

Medium Risk: ☐Use gel positioning pads ☐Apply eye ointment to pressure areas
☐Apply petroleum jelly to pressure areas ☐Wrap ETT, filters, etc., with laparoscopic camera sleeve
☐Apply eye ointment, petroleum jelly, or moisturizer to nasal area

High Risk: ☐Apply prophylactic dressings to pressure areas
☐Apply prophylactic dressings to pressure areas for diabetic patients

Intraoperative Pressure Injury Risk Factor Dynamic Assessment (Mark √ in ☐, Total Score: ______ points, ______ Risk)

Item & Assessment	1 point	2 point	3 point	4 point
Heat Loss Factors	Superficial tissue exposure ☐	Deep tissue exposure ☐	Body cavity/organ exposure ☐	Hypothermia/ Antipyretics ☐
Intraoperative Blood Loss (ml)	<200 ☐	≧200&<400 ☐	400-800 ☐	>800 ☐
Pressure/Shear Force Changes	Mild increase ☐	Moderate increase ☐	Severe increase ☐	Extreme increase ☐
Actual Surgery Time (h)	<3 ☐	≧3&<3.5 ☐	≧3.5&<4 ☐	≧4 ☐

Intraoperative Assessment:> 12 points = High-Risk; 8 - 12 points = Medium-Risk; <8 points = Low-Risk.

Nursing Interventions:

Low Risk:☐Comprehensive warming measures (blankets, limb wrapping, warmed irrigation fluids, adjust room temp)
☐Monitor blood loss & BP ☐Pressure relief (add cotton pads/gel pads to pressure areas)
☐Retrieve temporarily unused instruments ☐Monitor nasal skin condition

Medium Risk: ☐Active warming measures (surface warming, fluid/blood product warming)
☐In case of hypoperfusion events, follow orders: establish multiple IV lines, adjust infusion/transfusion rates
☐For patients with BMI>40, or surgery time>6h, or age>75 years, apply prophylactic dressings to pressure areas
☐Remind surgeon about appropriate suture tension for nasal/ETT fixation ☐Adjust ETT position
☐Remind surgeon not to suture-fix NG tube to ETT and to remove promptly post-op
☐Keep ETT suspended in nasal cavity as much as possible

High Risk:☐Alternating left/right side for occipital pressure
☐Adjust OR table tilt 5 - 10 degrees to shift pressure areas
☐Apply eye ointment, moisturizer, or povidone-iodine to nasal area during wound closure

Postoperative Pressure Area Skin Assessment (Mark √ in ☐)

☐Normal ☐Present-on-Admission Pressure Injury Location: __________________ Area: ________cm×_______cm

☐Intraoperative-Acquired Pressure Injury: ☐Non-blanchable erythema ☐Stage 1 ☐Stage 2 ☐Stage 3
☐Deep Tissue Injury ☐Unstageable ☐Stage 4

☐Medical Device-Related Pressure Injury ☐Mucosal Membrane Pressure Injury

Location:__________________ Area: ________cm×_______cm Skin sustained pressure duration: ______h

Note: BMI = Weight (kg) / Height $(m)^2$
